# Utility of clinical assessment, imaging, and cryptococcal antigen titer to predict AIDS-related complicated forms of cryptococcal meningitis

**DOI:** 10.1186/1742-6405-7-29

**Published:** 2010-08-03

**Authors:** Edward R Cachay, Joseph Caperna, Amy M Sitapati, Hamta Jafari, Sean Kandel, William C Mathews

**Affiliations:** 1 Department of Medicine, University of California San Diego, 200 W. Arbor Drive, San Diego, California 92103. USA; 2 Department of Medicine, Alameda County Medical Center , 15400 Foothill Boulevard San Leandro, California 94578. USA; 3 Department of Chemistry, University of California San Diego, 9500 Gilman Drive, La Jolla, California 92093-0303, USA

## Abstract

**Background:**

This study aimed to evaluate the prevalence and predictors of AIDS-related complicated cryptococcal meningitis. The outcome was complicated cryptococcal meningitis: prolonged (≥ 14 days) altered mental status, persistent (≥ 14 days) focal neurologic findings, cerebrospinal fluid (CSF) shunt placement or death. Predictor variable operating characteristics were estimated using receiver operating characteristic curve (ROC) analysis. Multivariate analysis identified independent predictors of the outcome.

**Results:**

From 1990-2009, 82 patients with first episode of cryptococcal meningitis were identified. Of these, 14 (17%) met criteria for complicated forms of cryptococcal meningitis (prolonged altered mental status 6, persistent focal neurologic findings 7, CSF surgical shunt placement 8, and death 5). Patients with complicated cryptococcal meningitis had higher frequency of baseline focal neurological findings, head computed tomography (CT) abnormalities, mean CSF opening pressure, and cryptococcal antigen (CRAG) titers in serum and CSF. ROC area of log_2 _serum and CSF CRAG titers to predict complicated forms of cryptococcal meningitis were comparable, 0.78 (95%CI: 0.66 to 0.90) vs. 0.78 (95% CI: 0.67 to 0.89), respectively (χ^2^, p = 0.95). The ROC areas to predict the outcomes were similar for CSF pressure and CSF CRAG titers. In a multiple logistic regression model, the following were significant predictors of the outcome: baseline focal neurologic findings, head CT abnormalities and log_2 _CSF CRAG titer.

**Conclusions:**

During initial clinical evaluation, a focal neurologic exam, abnormal head CT and large cryptococcal burden measured by CRAG titer are associated with the outcome of complicated cryptococcal meningitis following 2 weeks from antifungal therapy initiation.

## Background

Cryptococcal meningitis remains one of the leading causes of morbidity and mortality in patients with AIDS in resource limited settings [1]. Up to twenty percent of patients with cryptococcal meningitis have minimal central nervous symptoms at clinical presentation and early diagnosis of meningitis is facilitated by use of cerebrospinal fluid (CSF) cryptococcal antigen (CRAG) [2]. Cryptococcal antigen availability and use are variable in developing countries [1]. Over the last twenty years at the University of California, San Diego (UCSD), we occasionally cared for patients with minimal or no symptoms related to the central nervous system, high serum CRAG titer (as high as 1:65,536) and ultimately fatal HIV-associated cryptococcal meningitis. This observation prompted us to study whether serum and/or CSF CRAG titers alone or in combination with other baseline clinical parameters could be used to identify AIDS patients at risk for complicated forms of cryptococcal meningitis. The study aims were to (1) establish the prevalence of complicated cryptococcal meningitis in our clinical cohort, (2) identify a parsimonious set of clinical and laboratory predictors of complicated cryptococcal meningitis, and (3) to examine the operating characteristics of quantitative predictors of complicated cryptococcal meningitis.

## Methods

### Study design and population

This retrospective case series of HIV-infected adults experiencing a first episode of cryptococcal meningitis was approved by the UCSD Human Research Protection Program (project# 071931) and performed at UCSD Medical Center. All patients provided written informed consent for the collection of samples and subsequent analysis. This study was conducted according to the principles expressed in the Declaration of Helsinki.

Patients needed to be either antiretroviral naive or, if not naive, off antiretrovirals for at least eight weeks prior to diagnosis of cryptococcal meningitis. Patients needed to be off fluconazole or other systemic antifungals at least eight weeks prior to diagnosis of cryptococcal meningitis. Baseline characteristics included epidemiological, clinical, serological, microbiological and radiologic data for each patient collected within 48 hours of admission. Serum and CSF CRAG titers were obtained concurrently at the time of first lumbar puncture. Three reviewers independently completed case study forms for each patient (S.K., H.J. & E.R.C). In case of data disagreement, reconciliation was performed by two independent reviewers (J.C & A.M.S.).

### Definitions and study outcomes

First lumbar puncture was performed within 48 hours of hospital admission and before systemic antifungal therapy was initiated. Since CSF opening pressure as high as 28 cmH_2_0 has been reported in normal asymptomatic individuals in the general population [3], we defined intracranial hypertension for this study as CSF opening pressure ≥ 30 cm H_2_0. Complicated cryptococcal meningitis, our primary outcome, was defined by the presence of any of the following four criteria: (1) prolonged (≥ 14 days) altered mental status (Glasgow scale score less than 13), (2) persistent (≥ 14 days) focal neurological finding, (3) placement of surgical CSF shunt during their hospitalization and (4) death occurring 48 hours after admission and during hospital stay.

### Serology

We detected CRAG in serum and CSF using a commercial latex agglutination assay (CALAS; Meridian Bioscience Europe, Nice, France) which included pronase treatment according to manufacturer instructions [4]. At UCSD microbiology laboratory, samples that test positive for CRAG in serum or blood are routinely diluted until finding the highest dilution associated with a 2 + or greater agglutination reaction.

### Statistical analysis

Patients were categorized in two groups according to the presence or absence of complicated cryptococcal meningitis. Variables between meningitis groups were compared using t-tests and Fisher's exact tests for continuous and categorical values, respectively. Serum and CSF CRAG operating characteristics to predict complicated cryptococcal meningitis were estimated using receiver operating characteristic curve (ROC) analysis [5]. Log transformation was used for cryptococcal antigen titers (base 2). Association between quantitative variables was estimated using Spearman's rho. Variables associated with the outcome in bivariate analysis (p < 0.05) were entered into a multivariate logistic regression model to identify independent predictors of the outcome. For multivariate analysis intracranial hypertension was entered as a categorical variable because initial CSF opening pressure was not recorded for all patients. The opening pressure variable was coded as: ≥ 30 cm of H_2_0, < 30 cm of H_2_0, and not recorded. Two way interactions were explored. Analysis was performed using Stata version 11.0 (Stata Corp., College Station, Texas, USA).

## Results

Between 1 January 1990 and 31 August 2009, 156 patients were admitted with AIDS-related cryptoccocal meningitis at UCSD. Seventy four were excluded from the study: 40 had recurrent episodes of cryptococcal meningitis, 11 were taking antiretrovirals, 13 had no CRAG available, and 10 left against medical advice within three days of admission. Eighty-two patients with first episode of cryptococcal meningitis comprised the study population: 93% were male; 63% were non-white. By HIV transmission risk factor, 60% were men having sex with men and 11% were injection drug users.

Fourteen patients (17%) met criteria for complicated cryptococcal meningitis (death 5, prolonged altered mental status 6, focal neurologic findings 7, CSF surgical shunt placement 8), Table 1. All patients with complicated cryptococcal meningitis were treated with Amphotericin B deoxycholate (AmpBd) and 5-Fluorocytosine (5-FC) during the induction phase (or for as long as they survived) followed by oral fluconazole 800 mg during the consolidation phase. However four patients were treated with monotherapy during the first 48 hours of hospitalization (only fluconazole 2, and only AmpBd 2). The patients who received only fluconazole during the first 48 hours had no initial symptoms referable to the central nervous symptoms. All deaths occurred during the first week of hospitalization (median: day 6, range: day 4 to 7) and the patients who survived remain alive for at least 6 months after outpatient follow up. Most patients who required a CSF surgical shunt placement had the intervention done during their third week of hospitalization (median: day 21, range: day 5 to 30). The one patient that had a CSF shunt placement in the first week developed coma rapidly with signs of decortications after admission and had persistently elevated CSF opening pressures with no clinical improvement despite daily lumbar punctures. There was no difference in age, gender, race/ethnicity, HIV risk factor, CD4 cell count or HIV plasma load between patients with and without complicated cryptococcal meningitis (Table 2). On initial clinical evaluation, there was no difference in the proportion of patients with meningeal signs, altered mental status or seizures comparing patients with and without complicated cryptoccocal meningitis (Table 2). Patients with complicated cryptococcal meningitis had higher frequency of baseline focal neurological findings (50 vs. 5%, p = 0.0001), head computed tomography (CT) abnormalities (21 vs. 2%, p = 0.03) and mean values of CSF opening pressure, (43 vs. 27 cmH_2_0, p = 0.0001), Table 2. All patients in the present study underwent head CT evaluation except two who were in the uncomplicated group (80 out of 82). The criteria for head CT abnormalities included: (1) enlarged ventricles consistent with hydrocephalus, (2) cerebritis and (3) focal lesions with or without mass effect. In the complicated group 3 patients had abnormal head CT findings (cerebritis 3 and enlarged ventricles 1) whereas only one patient had head CT abnormalities in the uncomplicated group (focal lesion in basal ganglia without mass effect). The focal neurologic findings found at baseline in patients who had a complicated course were ocular nerve palsies 4, hearing loss 2, and blindness 1. Of note, all patients who died had normal Glasgow scale scores on admission.

**Table 1:** Distribution of Complicated Meningitis Outcome Components

Outcome	N = 14
Only persistent altered mental status	1
Only CSF shunting procedure	4
Persistent focal finding and CSF shunting	3
Persistent focal finding and death	1
Persistent altered mental status and death	2
Persistent altered mental status and persistent focal finding and CSF shunting	1
Persistent altered mental status and persistent focal finding and death	2

CSF, cerebrospinal fluid.

14 out 82 studied patients developed forms of AIDS-related cryptococcal meningitis.

**Table 2:** Demographic, Clinical and Laboratory Characteristics of Study Patients with AIDS-Related Cryptococcal Meningitis

	All patients with cryptococcal meningitis	Uncomplicated cryptococcal meningitis patients	Complicated cryptococcal meningitis patients	P value
	n = 82	n = 68	n = 14	
Age	38 (19 - 57)	38 (19 - 57)	36 (25-48)	0.54
Race/ethnicity				
White	30(36.7)	28(41.2)	2(14.3)	
Latino	31(37.8)	24(35.3)	7(50.00)	
Black	17(20.7)	13(19.1)	4(28.6)	0.17
Asian	2(2.4)	1(1.5)	1(7.1)	
Other	2(2.4)	2(2.9)	0	
HIV risk factor				
MSM	49 (59.8)	38(55.8)	11 (78.7)	
Heterosexual	18 (22.0)	17 (25.0)	1 (7.1)	
IVDA	13 ( 15.8)	11 (16.2)	2 (14.2)	0.51
Hemophilia	1 ( 1.2)	1 (1.5)	0	
Unknown/other	1 (1.2)	1 (1.5)	0	
CD4 cell count (× 10^6^/l)	39.4 (2-256)	38.7 (2-256)	42.7 (3-139)	0.77
HIV plasma load, log10 copies/ml ^a^	5.3 (4.1-6.2)	5.3 (4.1-6.2)	5.5 (4.8-5.9)	0.48
Meningeal signs^b^	12 (14.6)	8 (11.8)	4 (28.6)	0.21
Initial altered mental status(Glasgow scale ≤ 13)^b,^	21 (25.6)	15 (22.1)	6 (42.9)	0.18
Focal neurological findings^b^	10 (12.2)	3 (4.4)	7 (50)	0.0001
Seizures ^b^	5 (6.1)	3 (4.4)	2 (14.3)	0.20
CSF opening pressure (cmH20)^c^	30 (5-61)	26.9 (5-57)	43.4 (15-61)	0.0001
CSF				
wbc (/μl)	45.9 (0-500)	49.9 ( 0-500)	26.3 (0-210)	0.36
glucose(mg/dl)	41.5 (2-122)	40.7 ( 2-103)	45.8 (11-122)	0.34
protein (mg/dl)	77.2 (25-278)	77.9 (27-278)	73.9 ( 25-178)	0.79
CSF India ink positive	71 (88)	57 (85)	14 (100)	0.20
CSF culture positive	78 (98)	64 (97)	14 (100)	1.0
Blood culture positive for Cryptococcus species^d^	43 (75)	35 (75)	8 (80)	1.0
Baseline log 2 serum CRAG	11 (3-16)	11 (3-16)	14 (8-16)	0.001
Baseline log 2 CSF CRAG	10 (1-18)	9 (1-18)	13 (10-16)	0.001
Initial abnormal head CT^e^	4 (4.9)	1 (1.5)	3 (21.4)	0.03
**OUTCOMES**^**f**^				
Persistent (≥ 14 days) altered mental status	6 (7)	0	6 (43)	0.0001
Persistent (≥ 14 days) focal neurological findings	7(9)	0	7(50)	0.0001
Required CSF surgical shunt	8 (10)	0	8 (57)	0.0001
Death	5 (6)	0	5 (36)	0.0001

Values shown are mean (range) or number of patients (%). MSM, men who have sex with men; IVDA, Intravenous drug use; CSF, cerebrospinal fluid; CRAG, cryptococcal antigen; CT, computed tomography;

^a ^Results available for 50 patients, 44 with uncomplicated and 6 with complicated cryptococcal meningitis.

^b ^Symptoms assessed at the time of initial physical evaluation on the emergency department.

^c ^Measurements in 68 patients, 53 with uncomplicated and 15 with complicated cryptococcal meningitis.

^d ^Results available for 57 patients, 45 with uncomplicated and 12 with complicated cryptococcal meningitis.

^e ^Not performed in 2 patients with uncomplicated cryptococcal meningitis.

^f ^These are components of the definition of complicated meningitis

Patients with complicated forms of cryptococcal meningitis had higher baseline CRAG titers in serum and CSF (p = 0.001, Table 2). ROC, 95% confidence intervals (CI) area of log_2 _serum CRAG to predict complicated forms of cryptococcal meningitis was comparable to that of log_2 _CSF CRAG, 0.78 (95%CI:0.66 to 0.90) vs. 0.78 (95% CI:0.67 to 0.89), respectively (χ^2^, p = 0.95). The ROC areas to predict the outcome were similar for both CSF opening pressure and log_2 _CSF CRAG (ROC area difference 0.04 (95% CI -0.12 to 0.20, p = 0.64)). There was a significant correlation between log_2 _CSF CRAG and CSF opening pressure (Spearman rho = 0.42, p = 0.0003) and also between log_2 _serum CRAG and CSF opening pressure (Spearman rho = 0.31, p = 0.01).

In bivariate categorical analysis, complicated forms of cryptococcal meningitis were strongly associated with the presence of baseline focal neurological findings [odds ratio (OR) 21.7, 95% CI: 3.7-149.3, p = 0.00001], CSF opening pressure ≥ 30 cm H_2_0 (OR 4.3, 95% CI: 1.02-19, p = 0.02), log_2 _CSF CRAG titer (OR 1.5, 95% CI:1.1-1.9) and head CT abnormalities (OR 17.7, 95% CI: 1.2-944, p = 0.002). Multiple logistic regression models identified focal neurologic findings, log_2 _CSF antigen titer, and head CT abnormalities as independent predictors of complicated cryptococcal meningitis (Table 3). Values of CSF opening pressure were not available in 14 patients (one with complicated and 13 without complicated course). Logistic regression models yielded similar results when performed with and without patients with missing CSF opening pressure values. Although CSF opening pressure ≥ 30 cm H_2_0 was strongly associated with the outcome in bivariate analysis, this effect was not detected when controlling for baseline focal neurologic deficit, CT abnormality, and CSF antigen titer.

**Table 3:** Unadjusted and Adjusted Risk Factors for Developing Complicated Cryptococcal Meningitis within Two Weeks of Admission

Risk Factor	Unadjusted OR (95% CI)	p	Adjusted OR (95% CI)	p
Baseline focal neurologic findings	21.7(3.7-149.3)	.00001	17.2(2.6-114.9)	.003
CSF opening pressure^a^		**.04**		**.44**
≥ 30 cmH_2_0	4.3(1.2-15.1)	.03	1.9(0.36-10.7)	.44
Missing CSF opening pressure^b^	0.6(0.1-5.8)	.67	0.37(0.02-6.7)	.51
Baseline log_2 _CSF CRAG	1.5(1.1-1.9)	.004	1.5(1.1-2.2)	.02
Initial abnormal head CT	17.7(1.2-944)	.002	32.6(1.1-927.8)	.04

^a ^Reference < 30 cm H_2_0

^b^Fourteen patients have no baseline CSF opening pressure measurement

OR , Odds ratio; CI, Confidence interval; CSF, cerebrospinal fluid; CRAG, cryptococcal antigen; CT, computed tomography.

Model N = 80, ROC area 0.92, Hosmer-Lemeshow χ^2 ^p < 0.00001

## Discussion

The present study to assess AIDS patients at risk for complicated forms of cryptococcal meningitis found that: (1) focal neurologic deficit, CT imaging abnormality, and CSF CRAG at the time of initial hospital evaluation independently predict the outcome of complicated forms of cryptococcal meningitis following two weeks from antifungal therapy.;(2) Serum and CSF CRAG as measures of fungal burden were comparable in their ability to discriminate between those with and without outcome; (3) CSF CRAG and initial opening pressure were comparable in ROC discrimination and (4) CRAG (both serum and CSF) was moderately correlated with initial CSF opening pressure.

AIDS related cryptococcal meningitis (in the absence of immune reconstitution) is often clinically characterized by a massive fungal burden with minimal CSF pleocytosis but with elevated CSF pressure [6,7]. Intracranial hypertension results is consequence of inflammatory cells invading and disrupting the architecture of the arachnoid granulations which then facilitate blockage of CSF reabsorption at the arachnoid granulations by the fungal organism [8]. The present study showed that the fungal burden correlates with CSF pressure, as has been shown before by a clinical and a pathologic study [8,9]. Our study adds that this association is present irrespective of whether fungal burden is assessed using serum or CSF CRAG. CRAG and India ink are common markers of fungal burden [10,11]. In this study only CRAG was associated with complicated cryptococcal meningitis. Indian ink is widely available in developing countries whereas CRAG is not [1]. We believe that the enhanced diagnostic sensitivity of antigen testing over India ink as well as the prognostic value quantitative antigen measurement demonstrated in this and other studies provide further evidence to support wider availability of quantitative antigen testing in developing settings.

Although, having a baseline CSF opening pressure ≥ 30 cmH_2_0 was associated with complicated forms of cryptococcal meningitis in bivariate analysis, it was not significant in multivariate analysis. Nonetheless, the severity of intracranial hypertension at baseline has been associated with fatal outcomes within two weeks of initiation of therapy in some studies [12], but not in all [9]. This difference may be explained by a number of factors: (1) lack of statistical power to detect a meaningful biological difference; (2)in those studies where no association was found, patients were enrolled in an aggressive CSF pressure management protocol with frequent lumbar punctures if found to have intracranial hypertension at baseline [9]; and (3) the wide distribution of normal CSF opening pressure values in the general population [3] may preclude detection of an association between intracranial hypertension and complications of AIDS-related cryptococcal meningitis. Nevertheless, current guidelines recommend measurement of CSF pressure in every AIDS patient undergoing clinical evaluation for meningitis [13]. We acknowledge selection bias in ascertainment of initial opening pressure. It is clear from both bivariate and multivariate models that those with unrecorded opening pressure had a prognosis similar to those with measured opening pressures < 30 cm H_2_0. We also note that in our cohort almost thirty percent of patients who developed a complicated course had no focal neurologic findings and only minimal central nervous system referable symptoms at time of presentation.

Death is not the only relevant outcome of this opportunistic infection [14]. Our definition of complicated cryptococcal meningitis includes death but also incorporates two elements of long term morbidity: (1) persistently (≥ 14 days) abnormal neurologic exam either by altered mental status or focal neurologic findings, and (2) surgical intervention to control intractable intracranial hypertension.

This was an observational retrospective study and important limitations need to be recognized. First, fourteen patients had no baseline opening CSF pressure measurement. Among those who had no CSF pressure documented, all but one had an uncomplicated course. The main reasons for missing CSF pressure documentation included: technical challenges that arose during lumbar puncture while performed in the emergency room and the illness episode occurred between 1990 and 1995 when routine measurement of CSF opening pressure was not as widely accepted as currently. Second, certain variables (Table 1) have missing data: (1) HIV viral loads were missing in 39% of patients, most of them diagnosed when viral loads were not widely available for patient care; (2) fungal blood cultures were not available in 17% of patients, which is due to the observational and retrospective nature of the study.

## Conclusion

In summary, a focal neurologic exam, abnormal head CT, and large cryptococcal burden measured by CRAG assessed within 48 hours of admission are associated with the outcome of complicated forms of cryptococcal meningitis assessed 2 weeks from antifungal therapy initiation. These findings underscore the importance of quantitative CRAG testing in AIDS patients with suspected cryptococcal meningitis, particularly in resource limited settings where the burden of cryptococcal meningitis is high and access to this technology is limited.

## Competing interests

The authors declare that they have no competing interests.

## Authors' contributions

ERC carried out study design, data collection, statistical analysis and draft manuscript. HJ and SK performed data collection and filled case report forms. JC and AMS, carried out data reconciliation by chart reviews. WCM participated in every single step of study from conception, design, statistical analysis and drafting of manuscript. All authors review and approved final version of manuscript.
